# Mechanism of Xu Li's Experiential Prescription for the Treatment of EGFR-Positive NSCLC

**DOI:** 10.1155/2020/8787153

**Published:** 2020-03-12

**Authors:** Bin Xu, Haitao Zhang, Yuchao Wang, Shuran Huang, Li Xu

**Affiliations:** ^1^Nanjing University of Chinese Medicine, Nanjing 210023, China; ^2^Department of Respiratory Medicine, The Affiliated Brain Hospital of Nanjing Medical University (Chest Hospital District), Nanjing 210029, China; ^3^Department of Endoscopic Center, The Affiliated Brain Hospital of Nanjing Medical University (Chest Hospital District), Nanjing 210029, China; ^4^The Third Affiliated Hospital of Nanjing University of Chinese Medicine, Nanjing 210022, China; ^5^The First Clinical Medical College, Nanjing University of Chinese Medicine, Nanjing 210023, China

## Abstract

The non-small-cell lung cancer (NSCLC) is the most common lung cancer which seriously threatens the human health. Xu Li's experiential prescription (XLEP) can treat the NSCLC. However, whether XLEP can regulate the autophagy in the EGFR-positive NSCLC still remains unknown. We found that the cellular activity of drug-resistant cells and sensitive cells were all decreased in the TCM group and TCM + Gef group. The expression of autophagy-associated proteins (mTOR and Beclin1-Vps34) in drug-resistant cells was decreased in the TCM group, while the expression of autophagy-associated proteins in sensitive cells was all decreased in the TCM + Gef group. The ratio of M1/M2 macrophages was increased when IL-4-induced RAW264.7 was treated with TCM. TCM treatment promoted the expression of CCL2 and CCL3 while it downregulated the CCL22 level among A549, H1975, and PC9 cells. The expression of TNF-*α* and IL-6 was increased, and the expression of IL-10 and TGF-*β* was decreased in IL-4-induced RAW264.7 cells treated with TCM. And, TCM treatment also decreased the expression of Fizz1 and TGM2. In conclusion, this study indicated that XLEP could suppress the proliferation of EGFR-TKI-resistant cancer cells and increase the ratio of M1/M2 macrophages by inhibiting autophagy to treat the drug-resistant EGFR-positive NSCLC.

## 1. Introduction

Lung cancer is a common malignant tumor of the lung and threatens the human health seriously. The incidence of lung cancer in the world has been on the rise. According to the histopathological characteristics, lung cancer can be divided into small cell lung cancer (SCLC) and non-small-cell lung cancer (NSCLC) and NSCLC accounts for 80%–85% of lung cancer [1, 2].

The epidermal growth factor receptor (EGFR) is a receptor existing on the surface of human glial cells, epithelial cells, fibroblasts, and so on and plays a vital role in cell division and apoptosis, cell differentiation, cell migration, and organogenesis [3, 4]. High expression of EGFR combined with its ligand to form homologous dimer promotes the activation of tyrosine protein kinase. EGFR was overexpressed in many human cancers including ovarian, colorectal, and non-small-cell lung cancers [5–7]. At present, EGFR-TKI therapy has become an important treatment for EGFR-positive NSCLC but inevitably acquired resistance to EGFR-TKI after 6 to 12 months of treatment [8]. EGFR-TKI-induced changes in the autophagy of NSCLC are an important cause of drug resistance. Wang et al. [9] reported that erlotinib could induce autophagy in sensitive and resistant cells, and autophagy inhibition could enhance the damaging effect for NSCLC. Similar reports have been reported in EGFR-overexpressed tumors, such as breast cancer [10], plasmoblastoma [11], and head and neck squamous cell carcinoma [12]. These findings all support that autophagy is the mechanism of cell survival which contribute to the resistance of NSCLC to EGFR-TKI, and autophagy inhibition can enhance the sensitivity of tumor cells to drugs.

Traditional Chinese medicine (TCM) has been widely used and has been demonstrated to be effective in NSCLC treatment [13–15]. In the previous studies, Xu Li's experiential prescription (XLEP) was applied to the treatment of NSCLC [16, 17]. The main ingredients of XLEP are *Radix adenophorae, Radix Glehniae, Radix Asparagi, Radix Ophiopogonis, Schisandra, Privet fruit, Astragalus, Zedoary*, and so on. Ophiopogonin B extracted from *Radix Ophiopogonis* has the antihepatoma effect by regulating the Akt/mTOR signaling pathway and autophagy [18]. Schisandrin A from *Schisandra* has been demonstrated to activate autophagy and inhibit apoptosis to protect liver injury [19]. Astragalus polysaccharide, the important active ingredient of *Astragalus*, could reduce cardiomyocytes autophagy to improve cardiac function after injury induced by ischemic reperfusion [20]. On account of these, we speculated that XLEP could be useful for the regulation of autophagy in NSCLC. Autophagy is also involved in the production of TAMs in tumor microenvironment, including the process of macrophages polarizing into TAMs. The number of M2 macrophages in solid tumors was higher than that of M1 macrophage- and M2 macrophage-promoted angiogenesis and tumor growth [21, 22]. We speculated that the number of M2 macrophages was decreased by regulating the autophagy in macrophages to adjust the ratio of M1/M2 macrophages, which is expected to become a new antitumor treatment method.

The present study aimed to investigate the effect of XLEP regulating the autophagy to treat the drug-resistant EGFR-positive NSCLC and the underlying molecular mechanism involved. XLEP could be a valuable therapy for the treatment of EGFR-positive NSCLC.

## 2. Materials and Methods

We followed the methods of Chen et al. [23].

### 2.1. Preparation of XLEP

The compositions of XLEP were purchased from Shanghai LEY'S Pharmaceutical Co., Ltd. The contents of XLEP are as follows: 30 g *Adenophorae Radix*, 30 g *Radix Glehniae*, 30 g *Asparagus*, 30 g *Radix Ophiopogonis*, 10 g *Schisandra*, 30 g *Ligustrum lucidum*, 10 g *Turtle Shell*, 30 g *Astragalus membranaceus*, 30 g *Agrimonia pilosa*, 30 g *Oldenlandia*, 10 g *Duchesnea indica*, 15 g *Nightshade*, 10 g *Fritillaria thunbergii*, 10 g *Curcuma zedoary*, 10 g *Pheretima*, 10 g *Radix Glycyrrhizae*, 3 g *Panax pseudoginseng*. XLEP was prepared by decoction.

### 2.2. Preparation of Medicine Serum

Adult Sprague-Dawley (SD) rats (*n* = 10, half male and female) were allowed a 7-day adaptation period before starting experimental procedures. Then, SD rats were given by gavage twice a day (at 8 a.m. and 15 p.m.) with high-concentration Xu Li's experiential prescription (XLEP) (4-5 times equivalent dose) for two consecutive days. Within 1-2 hours after the last gavage, rats were anesthetized and killed by cervical dislocation. Blood was collected and centrifuged at 3000 rpm for 10 min at 4°C after 1-2 hours' standing. The serum was separated and stored at −80°C until further use. When the serum was used in the experiment, its temperature should be recovered at room temperature. The animal protocol was approved by the Laboratory Animal Care and Use Committee at Nanjing University of Chinese Medicine.

### 2.3. Cell Culture

Congenital EGFR-TKI drug-resistant A549 cell line, acquired EGFR-TKI drug-resistant H1975 cell line, and EGFR-TKI drug-susceptible PC9 cell line were obtained from the American Type Culture Collection (ATCC; Rockville, MD, USA) and were cultured in Dulbecco's modified Eagle's medium (DMEM; HyClone, Logan, UT, USA) containing 10% fetal bovine serum (FBS; Gibco, Gaithersburg, MD, USA) at 37°C in an atmosphere of 5% CO_2_. In the case of cells normal growth, cells passaged every 3-4 days. Cells in the logarithmic phase were divided into groups and intervened. The three kinds of cells were, respectively, divided into four groups: control group, Gef (Gefitinib) group, TCM (traditional Chinese medicine-containing serum) group, and Gef + TCM group. The cell suspension was adjusted to 6 × 10^4^ cells per milliliter and seeded into a 96-well plate with 80 *μ*L per well. In the control group, cells were without any treatment. In the Gef group, 20 *μ*L of 50 *μ*mol/mL gefitinib per well was added to a 96-well plate. In the TCM group, 20 *μ*L traditional Chinese medicine-containing serum per well was added into a 96-well plate. In the Gef + TCM group, 10 *μ*L traditional Chinese medicine-containing serum and 10 *μ*L of 100 *μ*mol/mL gefitinib per well were added into 96-well plate. Each well was repeated at least three times. The culture was conducted under normal conditions for 24 h.

### 2.4. CCK-8 Assay

After drug treatment for 24 h, cell proliferation was detected by the CCK-8 assay. A549, H1975, and PC9 cells were seeded in a 96-well plate (5,000 cells per well) and cultured with Dulbecco's modified Eagle's medium (DMEM) (Gibco, Grand Island, NY, USA) supplemented with 10% FBS at 37°C with 5% CO_2_ for 24 h. 10 *μ*L CCK8 solution was added to a 96-well plate incubated for 4 h. Finally, the OD value at 450 nm was detected by a Synergytm2 microplate reader (BioTek Instruments, Inc., Winooski, VT, USA).

### 2.5. Colony Formation Assay

After drug treatment for 24 h, cell suspension was prepared by 0.25% trypsin digestion and 1000 cells were seeded in the 60 mm cell culture dish which was cultured in an incubator at 37°C with 5% CO_2_. The culture medium was changed every three days. The culture was terminated when the visible clones appeared. The cells were fixed with methanol solution, stained with crystal violet, and observed and counted under an optical inverted microscope.

### 2.6. Western Blot Analysis

After culture for 24 h, cellular proteins and tissue proteins including EGFR, mTOR, Beclin1, and Vps34 were analyzed by the western blot analysis. A lysis buffer (Cell Signaling Technology, Inc., Danvers, MA, USA) containing protease inhibitors and phosphatase inhibitors was used to lyse the cells for the extraction of total proteins of cells. The protein concentration within the supernatant was determined with a BCA protein assay kit (Pierce; Thermo Fisher Scientifc, Inc.). 30 *μ*g protein per lane was subjected to 10% SDS-PAGE and transferred to polyvinylidene fluoride membranes (EMD Millipore, Billerica, MA, USA). These membranes were blocked with 5% skimmed milk powder at room temperature for 2 h, followed by the incubation with primary antibodies against EGFR, mTOR, Beclin1-Vps34, ATG14, p62, Fizz1, and TGM2 at 4°C overnight. Subsequent to washing with TBST buffer three times, membranes were incubated with anti-rabbit horseradish peroxidase-linked IgG secondary antibody at room temperature for 2-3 h (cat no. 7074; Cell Signaling Technology, Inc.; dilution, 1 : 2,000). Specific protein expression was normalized to GAPDH for total protein analyses. Finally, an enhanced chemiluminescence detection system (Super Signal West Dura Extended Duration Substrate, Thermo Fisher Scientifc, Inc.) was used to observe the protein bands. The blots were semiquantified by densitometric analysis (image lab Software, version 3.0 Beta 3).

### 2.7. M2 Polarization Model

RAW264.7 cells were treated by IL-4 to build the M2 polarization model. The RAW264.7 cells were, respectively, divided into four groups: control group, Gef group, Gef + TCM (low) group, and Gef + TCM (high) group. The RAW264.7 cells were seeded into a 96-well plate. In the control group, cells received no treatment. In the Gef group, 20 *μ*L of 50 *μ*mol/mL gefitinib per well was added to a 96-well plate. In the Gef + TCM (low) group, 10 *μ*L of 100 *μ*mol/mL gefitinib and 10 *μ*L traditional Chinese medicine-containing serum per well was added into a 96-well plate. In the Gef + TCM (high) group, 10 *μ*L of 100 *μ*mol/mL gefitinib and 20 *μ*L traditional Chinese medicine-containing serum per well were added into a 96-well plate.

### 2.8. Enzyme-Linked Immunosorbent Assay (ELISA)

The levels of CCL2, CCL3, and CCL22 in the culture supernatant of A549, H1975, and PC9 cells were, respectively, determined by ELISA using the human CCL2 ELISA kit, human CCL3 ELISA kit, and human CCL22 ELISA kit (Beyotime Biotechnology Co., Ltd., Shanghai, China). The levels of TNF-*α*, IL-6, IL-10, and TGF-*β* in the culture supernatant of RAW264.7 cells were, respectively, measured by using the human TNF-*α* ELISA Kit, human IL-6 ELISA kit, human IL-10 ELISA kit, and human TGF-*β* ELISA kit (Beyotime Biotechnology Co., Ltd.).

### 2.9. Immunofluorescence

Total IL-4-induced RAW264.7 cells were fixed in 4% formaldehyde for 15 min. After being washed with 0.01 M PBS for three times, cells were incubated with 10% normal goat serum for 30 min and then the serum was removed. Subsequently, cells were incubated against CD86 (M1 cell marker) and CD206 (M2 cell marker) at 4°C overnight. After PBS washing, corresponding secondary antibody was added to the cells which was then incubated at 37°C for 1 h. Cells were rinsed with PBS for 5 min and sealed with water-soluble sealant containing DAPI, which were observed and photographed under a fluorescent microscope.

### 2.10. Statistical Analysis

ANOVA was performed using SPSS16.0. Values are presented as mean ± SD. Statistical significance between groups was determined by Student's *t* test or one-way ANOVA test. *P* ≤ 0.05 was considered statistically significant.

## 3. Results

### 3.1. XLEP Affects the Proliferation of A549, H1975, and PC9 Cells

**Figure 1:**
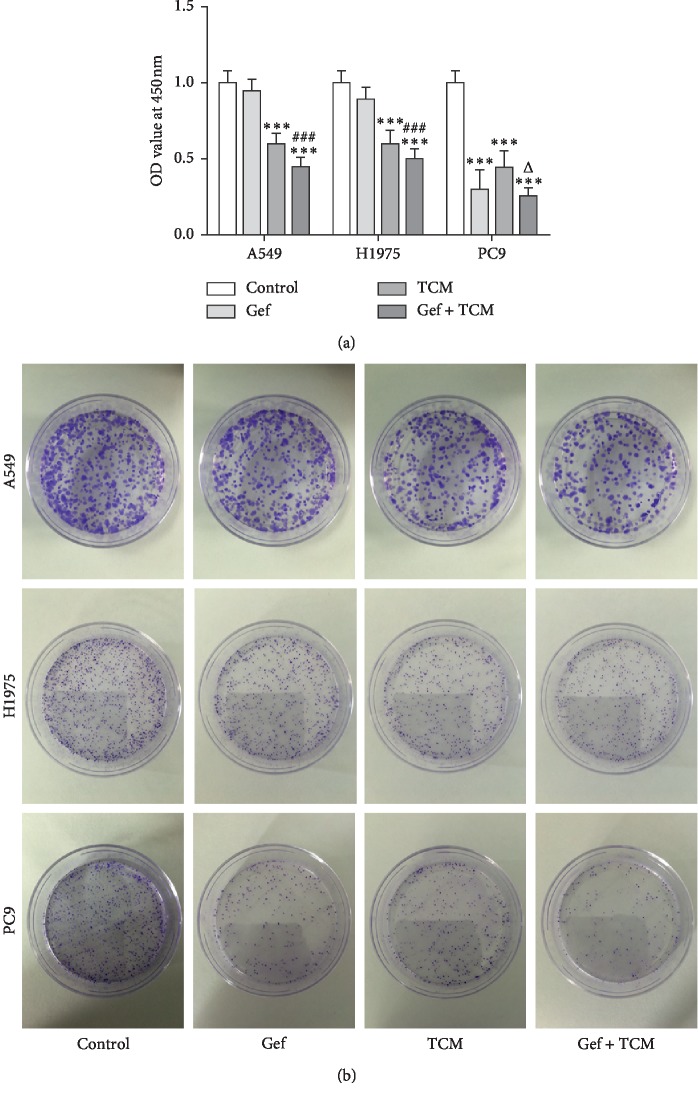
XLEP inhibits the proliferation of A549, H1975 and PC9 cells. (a) The viability of A549, H1975 and PC9 cells was analyzed by CCK-8 assay. ^*∗∗∗*^*P* < 0.001 vs. control group. ^###^*P* < 0.001 vs. Gef group. ^Δ^*P* < 0.05 vs. TCM group. (b) The proliferation of A549, H1975, and PC9 cells was determined by colony formation assay.

CCK-8 assay was applied to analyze the viability of A549, H1975, and PC9 cells (Figure 1(a)). The viability of PC9 cells was obviously inhibited in the Gef group compared with drug-resistant A549 and H1975 cells treated only with Gef where the viability showed a little change. And, the viability of these three cells was all obviously decreased in the group treated only with XLEP (TCM group) or gefitinib and XLEP (Gef + TCM group). The results of colony-formation assay showed the change of cell proliferation ability more directly (Figure 1(b)). Therefore, XLEP could inhibit the proliferation of PC9 cells and also effectively suppress the proliferation of drug-resistant A549 and H1975 cells.

### 3.2. XLEP Affects the Autophagy of A549, H1975, and PC9 Cells

**Figure 2:**
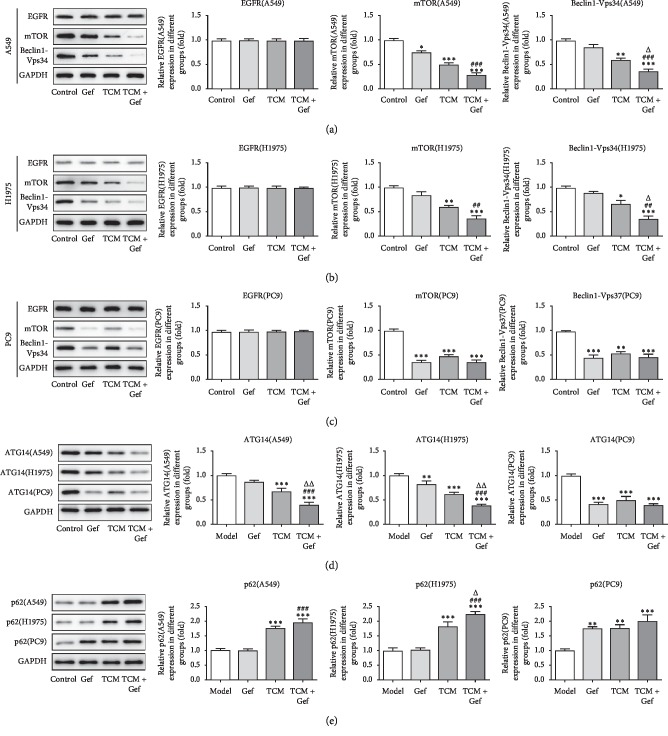
XLEP promotes the autophagy of A549, H1975 and PC9 cells. (a) The expression of EGFR, mTOR and Beclin1-Vps34 in A549 cells was detected by Western blot analysis. ^*∗*^*P* < 0.05, ^*∗∗*^*P* < 0.01 and ^*∗∗∗*^*P* < 0.001 vs. control group. ^###^*P* < 0.001 vs. Gef group. ^Δ^*P* < 0.05 vs. TCM group. (b) The expression of EGFR, mTOR and Beclin1-Vps34 in H1975 cells was detected by Western blot analysis. ^*∗*^*P* < 0.05, ^*∗∗*^*P* < 0.01 and ^*∗∗∗*^*P* < 0.001 vs. control group. ^##^*P* < 0.01 vs. Gef group. ^Δ^*P* < 0.05 vs. TCM group. (c) The expression of EGFR, mTOR, and Beclin1-Vps34 in PC9 cells was detected by western blot analysis. ^*∗∗*^*P* < 0.01 and ^*∗∗∗*^*P* < 0.001 vs. control group. (d) The ATG14 expression in A549, H1975, and PC9 cells was measured by western blot analysis. ^*∗∗*^*P* < 0.01 and ^*∗∗∗*^*P* < 0.001 vs. control group. ^###^*P* < 0.001 vs. Gef group. ^ΔΔ^*P* < 0.01 vs. TCM group. (e) The p62 expression in A549, H1975, and PC9 cells was measured by western blot analysis. ^*∗∗*^*P* < 0.01 and ^*∗∗∗*^*P* < 0.001 vs. control group. ^###^*P* < 0.001 vs. Gef group. ^Δ^*P* < 0.05 vs. TCM group.

The western blot was used to detect the expression of EGFR, mTOR, Beclin1-Vps34, ATG14, and p62 in A549, H1975, and PC9 cells (Figures 2(a)–2(e)). There are no obvious differences in the EGFR expression between four groups. The expression of mTOR, Beclin1-Vps34, and ATG14 in drug-resistant A549 and H1975 cells treated only with gefitinib was not obviously decreased, while that in PC9 cells was decreased significantly. The expression of mTOR, Beclin1-Vps34, and ATG14 in these three cells were all significantly downregulated in the TCM group and Gef + TCM group. The expression of p62 was obviously increased in A549, H1975, and PC9 cells treated only with XLEP (TCM group) or gefitinib and XLEP (Gef + TCM group). However, p62 expression was not obviously changed in A549 cells and H1975 cells treated only with gefitinib and increased in PC9 cells treated only with gefitinib. Therefore, XLEP promoted the autophagy of A549, H1975, and PC9 cells.

### 3.3. XLEP Regulates the Expression of Chemotactic Factors in A549, H1975, and PC9 Cells

**Figure 3:**
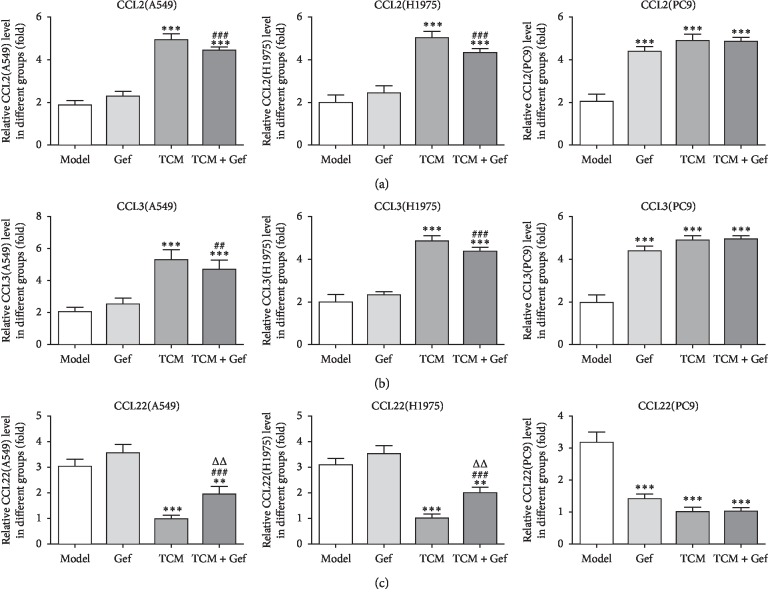
XLEP regulates the expression of chemotactic factors in A549, H1975, and PC9 cells. (a) The CCL2 level in the culture supernatant of A549, H1975, and PC9 cells was determined by ELISA. ^*∗∗∗*^*P* < 0.001 vs. control group. ^###^*P* < 0.001 vs. Gef group. (b) The CCL3 level in the culture supernatant of A549, H1975, and PC9 cells was determined by ELISA. ^*∗∗∗*^*P* < 0.001 vs. control group. ^##^*P* < 0.01 and ^###^*P* < 0.001 vs. Gef group. (c) The CCL22 level in the culture supernatant of A549, H1975, and PC9 cells and the culture supernatant of weredetermined by ELISA. ^*∗∗*^*P* < 0.01 and ^*∗∗∗*^*P* < 0.001 vs. control group. ^###^*P* < 0.001 vs. Gef group. ^ΔΔ^*P* < 0.01 vs. TCM group.

The chemotactic factors in cells and cell supernatant were detected with ELISA. The expression of CCL2 and CCL3 was increased in PC9 cells treated only with Gef while that showed no obvious change in drug-resistant A549 and H1975. However, the expression of CCL2 and CCL3 in these three cells treated only with XLEP (TCM group) or gefitinib and XLEP (Gef + TCM group) was increased remarkably compared with the control group (Figures 3(a) and 3(b)). The CCL22 expression was decreased in PC9 cells treated only with Gef, while that showed no obvious change in drug-resistant A549 and H1975. However, the CCL22 expression was downregulated in these three cells treated only with XLEP (TCM group) or gefitinib and XLEP (Gef + TCM group) (Figure 3(c)).

### 3.4. XLEP Regulates the Ratio of M1/M2 Macrophages

**Figure 4:**
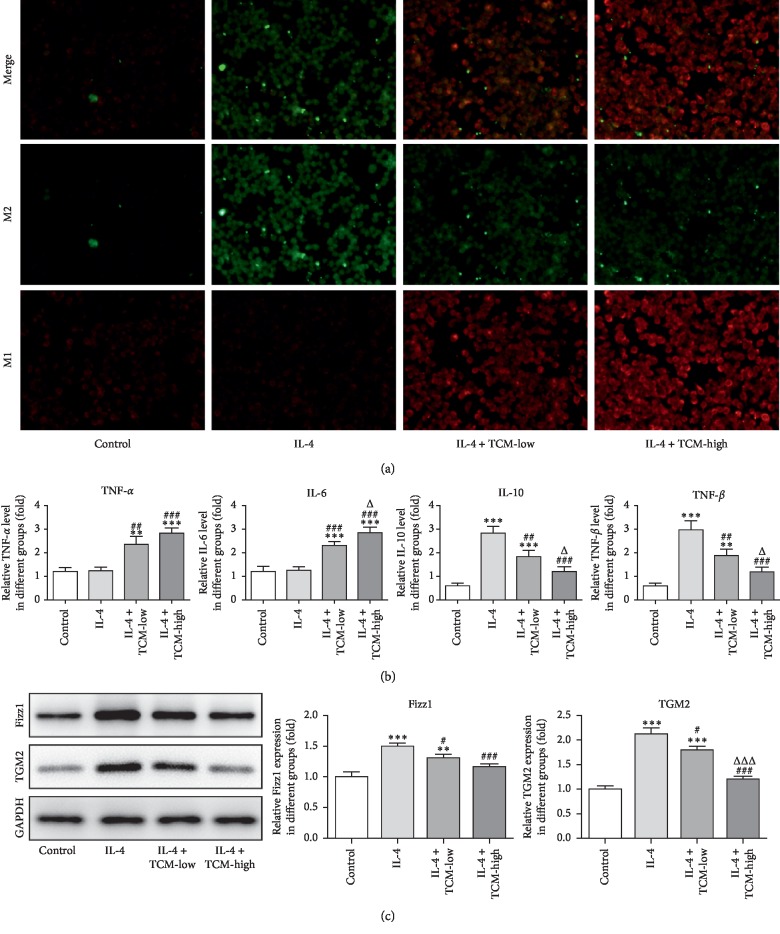
XLEP upregulates the ratio of M1/M2 macrophages. (a) The number of M1 and M2 macrophages was determined by immunofluorescence. (b) The levels of TNF-*α*, IL-6, IL-10, and TGF-*β* in the culture supernatant of RAW264.7 cells were detected by ELISA. ^*∗∗*^*P* < 0.01 and ^*∗∗∗*^*P* < 0.001 vs. control group. ^##^*P* < 0.01 and ^###^*P* < 0.001 vs. IL-4 group. ^Δ^*P* < 0.05 vs. IL-4 + TCM-low group. (c) The expression of Fizz1 and TGM2 in RAW264.7 cells was detected by western blot analysis. ^*∗∗*^*P* < 0.01 and ^*∗∗∗*^*P* < 0.001 vs. control group. ^##^*P* < 0.05 and ^###^*P* < 0.001 vs. IL-4 group. ^ΔΔΔ^*P* < 0.001 vs. IL-4 + TCM-low group.

Macrophage typing was detected by flow cytometry analysis. The ratio of M1/M2 TAMs was decreased in IL-4-induced RAW264.7 cells compared with that of the control group. And, the ratio of M1/M2 macrophages was gradually increased in the IL-4 induced RAW264.7 cells treated with low XLEP and high XLEP, which was tending to the ratio of M1/M2 macrophages in the control group (Figure 4(a)). As shown in Figure 4(b), TCM treatment promoted the expression of TNF-*α* and IL-6 in IL-4-induced RAW264.7 cells while TCM treatment downregulated the expression of IL-10 and TGF-*β* in IL-4-induced RAW264.7 cells. TNF-*α* and IL-6 were mainly secreted by M1 macrophages, and IL-10 and TGF-*β* were mainly secreted by M2 macrophages. The expression of Fizz1 and TGM2 was increased in IL-4 induced RAW264.7 cells, and XLEP obviously downregulated the expression of Fizz1 and TGM2 (Figure 4(c)). Therefore, XLEP could decrease the number of M2 macrophages and upregulated the ratio of M1/M2 macrophages.

## 4. Discussion

Here, we have investigated the role of XLEP regulating the autophagy in A549, H1975, and PC9 cells and the underlying molecular mechanism involved. It was shown that XLEP could inhibit the autophagy in A549, H1975, and PC9 cells and upregulated the ratio of M1/M2 macrophages to reduce the promotion effects of M2 macrophages on tumor cells. And, XLEP could be a valuable therapy for the treatment of EGFR-Positive NSCLC.

Lung cancer is a malignant tumor with high morbidity and mortality. According to the epidemiological survey, there are about one million new lung cancer cases in the world every year, in which about 210,000 cases in China and about 600,000 deaths a year from lung cancer, which poses a great threat on human life and health [24]. NSCLCs are the main kind in the lung cancer, and EGFR-positive NSCLC is a common type in the NSCLC. Currently, EGFR tyrosine kinase inhibitor (EGFR-TKI) is available in the treatment of EGFR-positive NSCLC, but congenital and acquired EGFR-TKI resistance seriously restricts the role of this class of drugs. Many studies have shown that EGFR-TKI induces protective autophagy for the treatment of NSCLC, leading to acquired drug resistance and disease recurrence [25, 26]. Therefore, it is possible to overcome the resistance of EGFR-TKI by inhibiting autophagy and improve the therapeutic effect of targeted drugs. In our study, the results showed that gefitinib as EGFR-TKI had no inhibiting effect on the proliferation and autophagy of A549 and H1975 while XLEP could inhibit the proliferation and autophagy of A549 and H1975.

CCL2 and CCL3 are secreted by M1 macrophages which promoted a strong immune response to kill the cancer cells [27, 28]. CCL22 is secreted by M2 macrophages which promoted tumor development and migration of cancer cells [29, 30]. Therefore, it might be useful to adjust the ratio of M1/M2 macrophages for the treatment of EGFR-TKI-resistant cancer cells. At present, we found that the expression of CCL2 and CCL3 was increased when the A549 and H1975 cells were treated with XLEP or gefitinib and XLEP. And, CCL2 and CCL3 were increased in PC9 cells treated with gefitinib or XLEP or gefitinib and XLEP. However, the variation tendency of CCL22 expression in A549, H1975, and PC9 cells was opposite to that of CCL2 and CCL3. In this study, we have constructed the RAW264.7 M2 polarization model induced by IL-4. After the treatment of XLEP, the ratio of M1/M2 macrophages was increased compared with the IL-4 induced RAW264.7 M2 polarization and the ratio of M1/M2 macrophages treated from low XLEP to high XLEP was tending to that in the control group. From above, XLEP could regulate the ratio of M1/M2 macrophages to decrease the generation of the M2 macrophages.

The high expression of CXCL10, TNF-*α*, and IL-1*β* in M1 macrophages can be used as molecular markers of M1 macrophages [31], and M1 macrophages decreased the secretion of IL-6, TNF-*α*, and IL-1*β* [32]. The expression of CD206, TGF-*β*, and IL-10 is highly expressed in M2 macrophages [33, 34]. IL-4 can promote the expression of CD206 in macrophages [35, 36]. We found that TCM treatment promoted the expression of TNF-*α* and IL-6 in IL-4-induced RAW264.7 cells, while TCM treatment downregulated the expression of IL-10 and TGF-*β* in IL-4-induced RAW264.7 cells. One study reported that curcumin could increase the expression of KLF4, FIZZ, and MGL1 in LPS-induced RAW264.7 cells, indicating that curcumin could promote the polarization of M1 macrophages to M2 macrophages [37]. Mouse and human M2 macrophages expressed TGM2 [38]. In this study, the expression of Fizz1 and TGM2 was decreased when IL-4-induced RAW264.7 cells treated with TCM. The above changes are consistent with the results of immunofluorescence.

In conclusion, results of this study demonstrate that XLEP can suppress the proliferation and autophagy of EGFR-TKI-resistant cancer cells to solve the problem that EGFR-TKI-resistant cancer cells is insensitive to gefitinib. In addition, XLEP can upregulate the ratio of M1/M2 macrophages by inhibiting autophagy to achieve the therapeutic effect for drug-resistant EGFR-positive NSCLC.

## Data Availability

The experimental data would be provided by the corresponding author upon request.
